# Triple-negative breast cancer: current understanding and future perspectives

**DOI:** 10.37349/etat.2026.1002372

**Published:** 2026-05-14

**Authors:** Alhasan Alobaidi, Vikrant Rai

**Affiliations:** IRCCS Istituto Romagnolo per lo Studio dei Tumori (IRST) “Dino Amadori”, Italy; Department of Translational Research, College of Osteopathic Medicine of the Pacific, Western University of Health Sciences, Pomona, CA 91766, USA

**Keywords:** triple-negative breast cancer, tumor heterogeneity, immunotherapy, therapeutic challenges

## Abstract

Triple-negative breast cancer (TNBC) is an aggressive breast cancer subtype defined by the absence of estrogen receptors, progesterone receptors, and human epidermal growth factor receptor 2 (HER2) expression. Consequently, standard hormone and HER2-targeted therapies are ineffective, necessitating reliance on chemotherapy, immunotherapy, antibody-drug conjugates (ADCs), and poly(ADP-ribose) polymerase (PARP) inhibitors for *BRCA*-mutated cases. TNBC exhibits rapid growth, a high risk of early recurrence, and disproportionately affects younger women, Black women, and *BRCA1* mutation carriers. Standard management typically involves neoadjuvant chemotherapy followed by surgery and potential radiation. However, TNBC treatment remains challenging due to its severe biological heterogeneity, high metastatic potential, and the toxicity associated with systemic therapies. This review discusses the current understanding of TNBC biology, highlighting the urgent need for advanced diagnostics, integrated molecular subtyping, and personalized targeted therapies.

## Introduction

Breast cancer (BC), a pathological condition, usually originates from the milk duct (ductal carcinoma) or, less commonly, from the lobules (lobular carcinomas), and is characterized by the uncontrolled growth of abnormal cells in the epithelial layers of breast tissue [1, 2]. It is the most common malignant tumor among women worldwide and is the leading cause of mortality among women globally, accounting for approximately one in eight cancer cases [2, 3]. BC remains one of the most commonly diagnosed cancers worldwide and is the second leading cause of cancer-related death among women in the United States [2, 4–6]. The high mortality is compounded by the fact that, despite global advancements in early detection and treatment, progress in managing advanced stages remains limited [2, 7]. Overall, the disease’s substantial global burden is reflected in the estimated 2.3 million new BC diagnoses and 670,000 BC-related deaths worldwide in 2022. Looking forward, the global incidence of BC is projected to reach 3.2 million cases by 2050, representing a 38% increase, while BC-related deaths are expected to rise by 68% to an estimated 1.1 million globally [8].

The molecular classification of BC is predicated upon the expression profiles of the estrogen receptor (ER), progesterone receptor (PR), and human epidermal growth factor receptor 2 (HER2), defining four principal intrinsic subtypes: luminal A, luminal B, HER2-enriched, and triple-negative BC (TNBC) [2, 4, 6, 9–12] (Table 1).

**Table 1:** Classification of breast cancer by molecular gene expression profiles.

**Subtype**	**Receptor status/Defining features**	**Prognosis and key characteristics**
Luminal A [2, 10, 11]	ER+/PR+/HER2–, HR+/HER2–. Characterized by low Ki-67 proliferation marker and generally low grade.	Most common subtype (up to 68% of all cases). Associated with a favorable prognosis.
Luminal B [2, 10, 11]	ER+, PR+/–, HER2–/+. Characterized by HR+/HER2+ and/or high Ki-67 expression. Approximately 10–30% of cases.	Poorer prognosis compared to luminal A. Exhibits aggressive behavior. Higher histological grade.
HER2-enriched (HER2+) [2, 4, 10, 11]	ER–/PR–/HER2+. Defined by HER2 overexpression or *ERBB2* gene amplification. Constitutes 12–20% of cases.	High Ki-67 expression, indicating aggressive behavior.
TNBC (basal-like) [1, 4, 7, 13]	ER–/PR–/HER2–. Lacks expression of all three receptors/markers. Overlaps significantly with the basal-like molecular subtype (about 80% of TNBC cases).	Most aggressive subtype. High histological grade. Associated with higher recurrence, high metastatic potential, and dismal prognosis/poor overall survival.

This classification is crucial because the different subtypes have distinct oncogenic drivers, leading to significant variations in clinical outcomes, tumor heterogeneity, and responsiveness to specific therapeutic interventions. The molecular heterogeneity within TNBC is further elucidated by subtyping systems (e.g., Lehmann, Burstein), which identify specific targetable pathways (e.g., luminal androgen receptor (LAR), immunomodulatory (IM), mesenchymal (M), and basal-like [BL1/BL2]) to guide precision medicine efforts [4, 11, 14, 15]. ER: estrogen receptor; PR: progesterone receptor; HER2: human epidermal growth factor receptor 2; ERBB2: Erb-B2 receptor tyrosine kinase 2 (also known as HER2); TNBC: triple-negative breast cancer.

While both luminal A and luminal B subtypes are hormone receptor (HR)-positive, they are primarily stratified clinically by the expression of the Ki-67 proliferation index. Luminal A tumors are typically characterized by low Ki-67 expression (defined as < 14%), indicating a lower proliferation rate and a more favorable prognosis, whereas luminal B tumors present with higher Ki-67 levels (≥ 14%). This denotes highly proliferative cells, a higher histological grade, and more aggressive clinical behavior [16].

These subtypes exhibit fundamentally distinct oncogenic drivers and thus require differential therapeutic approaches [2, 4, 5, 9, 17]. Luminal A and B cancers (HR-positive) are driven by hormonal signaling pathways, whereas the HER2-enriched subtype is characterized by the amplification or overexpression of the *ERBB2* gene [4, 6, 10, 11]. TNBC is defined by the absence of ER, PR, and HER2 expression (ER–PR–HER2–) and accounts for roughly 10 to 20 percent of BC cases [4, 6, 7, 9, 11, 13–15, 17]. This subtype is distinguished by its aggressive clinical behavior, high histologic grade, rapid proliferation, and early propensity for visceral and central nervous system metastasis [4, 6, 13]. TNBC also experiences an early peak in recurrence, most often within five years of diagnosis, and is consistently associated with shorter overall survival (OS) compared with other BC subtypes [4–6, 9, 13, 14]. The lack of targetable receptors renders TNBC unresponsive to endocrine therapy or HER2-targeted treatments, leaving cytotoxic chemotherapy as the historical backbone of management [5, 11, 13, 14, 17].

The reason no single, standardized “targeted therapy” exists for the entire TNBC population is rooted in the disease’s pronounced biological and molecular heterogeneity [5, 13, 17]. TNBC is an umbrella term covering diverse and heterogeneous subtypes of BC [5], encompassing multiple distinct molecular subtypes [e.g., basal-like, mesenchymal, luminal androgen receptor (LAR)] driven by diverse and complex oncogenic pathways rather than a singular dominant receptor [18]. This intrinsic complexity means that a single drug cannot target all TNBC tumors effectively [15, 19], limiting targeted interventions to small, biomarker-defined subgroups [5, 15, 17].

While chemotherapy has historically been the sole systemic option due to the lack of targetable receptors, recent breakthroughs have established highly effective, precision-driven approaches for treating TNBC. Currently, the most effective therapeutic strategies integrate immune checkpoint inhibitors (ICIs, such as pembrolizumab) with neoadjuvant chemotherapy (NACT) for early-stage high-risk disease, utilize targeted poly(ADP-ribose) polymerase (PARP) inhibitors for patients harboring germline *BRCA1/2* mutations, and employ antibody-drug conjugates (ADCs) [such as sacituzumab govitecan (SG)] to overcome chemoresistance in the metastatic setting [5, 20].

## Biology and pathogenesis

The aggressive clinical nature of TNBC is directly rooted in its distinct intrinsic molecular landscape, characterized by pervasive genomic instability and frequent pathogenic mutations in key regulatory pathways (high incidence of *BRCA1/2* mutations) [13, 15, 18]. At the molecular level, TNBC tumors exhibit a significantly high tumor mutational burden (TMB) and genomic rearrangement rates compared to other BC subtypes [13, 21]. The most common somatic alteration in TNBC is mutation of the tumor suppressor gene *TP53*, with mutation rates frequently reported between 60% and 88% [12, 22]. Other frequently altered pathways and oncogenes include phosphatidylinositol-3 kinase (PI3K)/protein kinase B (AKT)/mammalian target of rapamycin (mTOR) and mitogen-activated protein kinase (MAPK) signaling, which drive proliferation and survival of tumor cells, as well as the overexpression of epidermal growth factor receptor (EGFR) in a high percentage of tumors [1, 4, 13].

A critical genetic vulnerability that defines a major subset of TNBC is functional deficiency in DNA repair pathways, specifically those related to the *BRCA1* and *BRCA2* tumor suppressor genes [4, 21]. Germline pathogenic variants in *BRCA1/2* are strongly linked to the development of TNBC, occurring in approximately 10% to 30% of cases. TNBC accounts for nearly 60% of BCs in premenopausal women carrying *BRCA1* mutations [4, 21]. BRCA proteins are essential components of the homologous recombination repair (HRR) pathway, which is responsible for accurately fixing DNA double-strand breaks (DSBs) [4, 11]. The resulting homologous recombination deficiency (HRD) in BRCA-mutated TNBC creates a therapeutic weakness that can be exploited by PARP inhibitors [4, 6, 18]. PARP inhibitors block the repair of single-strand breaks (SSBs); in the context of concurrent HRD, the resulting accumulation of lethal DSBs leads to cancer cell death via synthetic lethality, making PARP inhibitors like olaparib and talazoparib effective targeted therapies for this specific patient subgroup [4]. The reliance of TNBC on complex, non-receptor-driven pathways (such as PI3K/AKT/mTOR and TP53) and its status as a heterogeneous collection of diseases historically limited systemic treatment to conventional cytotoxic chemotherapy [18].

The tumor microenvironment (TME) is a dynamic ecosystem that governs the aggressive behavior and therapeutic resistance of TNBC [7]. This extrinsic component consists of the extracellular matrix (ECM), vasculature, cancer-associated fibroblasts (CAFs), and tumor-associated macrophages (TAMs) [7, 10, 23]. Pathologic remodeling of the ECM leads to dense, stiff stroma that actively promotes tumor progression, invasion, and metastasis [7, 24]. Tumor stroma acting as a physical barrier restricts the effective penetration and distribution of chemotherapeutic agents, thereby diminishing treatment efficacy [7, 10]. Furthermore, stromal cells are reprogrammed to drive malignancy. CAFs proliferate and suppress antitumor immunity by secreting growth factors and inflammatory cytokines. TAMs, often polarized into the M2-like pro-tumor phenotype, represent the most abundant immune cell population and actively promote angiogenesis, metastasis, and drug resistance within this immunosuppressive niche [3, 7].

TNBC is the most immunogenic subtype, exhibiting higher TMB, which generates tumor-specific neoantigens, and a pronounced infiltration of immune cells, particularly tumor-infiltrating lymphocytes (TILs) [5, 21, 23]. These high TIL levels are strongly correlated with improved outcomes, including higher pathological complete response (pCR) rates after chemotherapy, and elevated expression of immune checkpoints like programmed death ligand 1 (PD-L1) [5, 6, 12]. This immunogenic profile provides a strong rationale for ICI therapy, which aims to unleash the host’s anti-tumor T-cell response [13, 14]. However, the immune response is often profoundly suppressed by the TME, which actively recruits regulatory T cells (Tregs) and M2-like TAMs, creating a cold or immunosuppressive landscape that blunts cytotoxic T lymphocyte (CTL) function [19, 23]. Consequently, despite the potential, clinical outcomes remain heterogeneous, and durable responses to ICIs are restricted to a subset of patients, necessitating sophisticated stratification strategies to overcome this complexity and realize the potential of precision immunotherapy [13, 15, 19]. This complexity means that single-agent targeted therapies, including immunotherapies, are not universally effective, limiting precision interventions to specific biomarker-defined subgroups and underscoring the need for sophisticated stratification strategies [13, 17, 19].

## Current management and limitations

**Figure 1:**
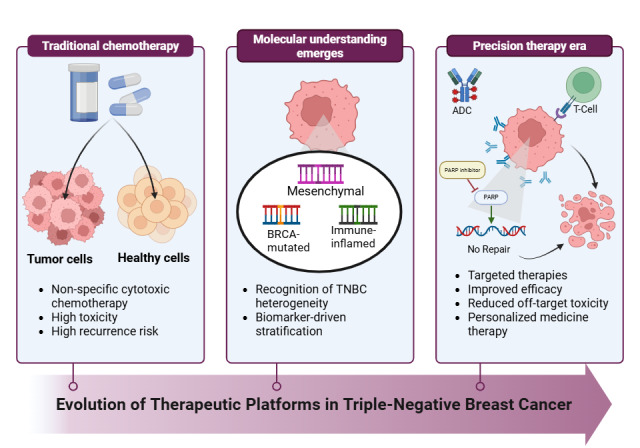
**Evolution of therapeutic platforms in triple-negative breast cancer (TNBC).** Schematic illustrating the progression of TNBC treatment from non-specific chemotherapy toward biomarker-driven precision therapies, including immunotherapy, poly(ADP-ribose) polymerase (PARP) inhibitors, and antibody-drug conjugates (ADCs). The therapeutic landscape for TNBC has shifted from solely relying on chemotherapy to a personalized, multi-platform approach. Key advancements since 2018 include the integration of immune checkpoint inhibitors, ADCs, and PARP inhibitors. These innovations have significantly improved pathological complete response (pCR) rates in early-stage disease and offered new treatment options for advanced/metastatic stages. Created in BioRender. Alobaidi, A. (2026) https://BioRender.com/xx583es.

The management of TNBC relies on an intensive, multimodal treatment strategy centered primarily on systemic therapy [1, 5, 18]. The current standard of care for non-metastatic TNBC integrates surgery, radiotherapy, and systemic therapy, with a strong preference for administering systemic therapy [4, 18] (Figure 1).

**Figure 2:**
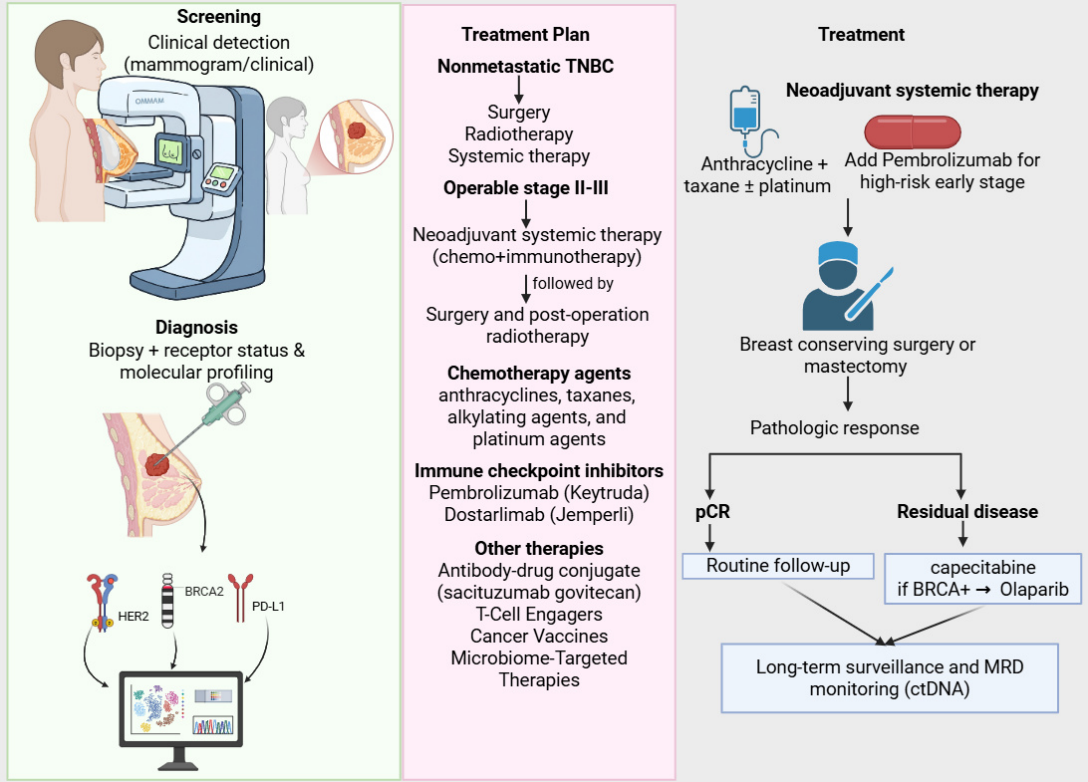
**Clinical treatment flow for early-stage triple-negative breast cancer (TNBC).** After diagnostic biopsy and molecular profiling, most patients proceed to neoadjuvant systemic therapy (chemotherapy with or without immunotherapy), followed by surgery. Pathologic complete response is associated with a favorable prognosis, while residual invasive disease prompts adjuvant escalation strategies including capecitabine, olaparib for germline *BRCA* carriers, or enrollment in trials with antibody-drug conjugates (ADCs) or combination regimens. Long-term surveillance increasingly includes ctDNA-based MRD monitoring. HER2: human epidermal growth factor receptor 2; MRD: minimal residual disease; pCR: pathological complete response; PD-L1: programmed death ligand 1. Created in BioRender. Alobaidi, A. (2025) https://BioRender.com/kkefeic.

For operable stage II–III TNBC, neoadjuvant systemic treatment (NAST), typically chemotherapy combined with immunotherapy, is the preferred approach, followed by surgery and post-operative radiation therapy [4–6] (Figure 2). Chemotherapy is the backbone of TNBC management because endocrine therapy and anti-HER2 targeted monoclonal antibodies are ineffective [4, 5, 11]. Standard chemotherapy regimens include combinations of anthracyclines, taxanes, alkylating agents, and sometimes platinum agents [18, 25] (Figure 2). The contemporary treatment landscape has evolved to include precision therapies for specific patient subsets [5, 13, 24]. ICIs are integrated with neoadjuvant chemo-immunotherapy standard for high-risk early-stage TNBC [4, 6]. PARP inhibitors like olaparib are used in patients with germline *BRCA1/2* mutations [4, 13, 22].

The main limitations of cytotoxic chemotherapy include its non-specific action, causing systemic toxicity and significant side effects, and the high rate of acquired resistance and relapse that follows initial responsiveness [18, 26]. This propensity for relapses is primarily driven by the disease’s innate molecular heterogeneity [19] and evolutionary dynamics, where cytotoxic chemotherapy acts as a selective pressure, favoring the survival and expansion of pre-existing, chemo-resistant cell populations [19]. NACT is effective in inducing a response, with pCR rates ranging from approximately 30–40% with traditional regimens [6, 12], increasing significantly (up to 40–65%) when platinum agents or ICIs are added [6, 25]. Achieving pCR is of critical prognostic importance [5, 6, 9]. Patients who attain pCR experience excellent long-term outcomes and improved survival rates [4–6]. Conversely, those with residual invasive disease (pathologic incomplete response, or pIR) face an unacceptably high risk of early tumor recurrence and significantly worse survival outcomes [4, 5].

The integration of immunotherapy into the TNBC treatment paradigm represents a fundamental shift away from chemotherapy as the sole systemic option, transforming the disease landscape toward precision oncology [5, 17, 19]. As a class of monoclonal antibodies that block immune checkpoints (e.g., PD-1/PD-L1), ICIs unleash the host’s anti-tumor T-cell response, providing a mechanism for durable, long-lasting clinical benefit previously unseen in this aggressive subtype. The most definitive proof of impact came from pivotal trials such as KEYNOTE-522, where the addition of the anti-PD-1 agent pembrolizumab to NACT achieved statistically significant improvements in both pCR rates and 3-year event-free survival (EFS) for patients with high-risk, early-stage TNBC [5, 13]. This advancement has notably improved long-term outcomes, especially for patients achieving pCR, a critical surrogate marker for survival [6, 13]. Immunotherapy is now a standard of care for specific patient groups across disease stages [13, 27]. In metastatic TNBC, pembrolizumab combined with chemotherapy is approved for unresectable locally recurrent or metastatic tumors with high PD-L1 expression (combined positive score ≥ 10) [13, 15, 22].

In contrast, for early-stage high-risk disease (stage II–III), pembrolizumab with NACT followed by adjuvant monotherapy is approved regardless of PD-L1 status [5, 6, 25], underscoring its broad utility in curative treatment. Atezolizumab previously had accelerated approval for PD-L1-positive metastatic TNBC, but this indication was later withdrawn [28, 29], reflecting ongoing refinement of effective immunotherapy agents and combinations. Despite these breakthroughs, not all TNBC patients respond to current immunotherapies [15, 23]. The varying molecular subtypes of TNBC mean a single targeted approach lacks universal efficacy. This inherent complexity underscores the need for comprehensive biomarker panels to accurately predict therapeutic benefit [5, 6, 13, 15, 17]. Table 2 summarizes the five primary tumor-intrinsic subtypes recognized in the refined Lehmann classification [4, 11], highlighting their molecular characteristics and corresponding therapeutic vulnerabilities derived from the literature [4, 11, 19].

**Table 2:** Lehmann classification (Vanderbilt Classification) of TNBC subtypes.

**Subtype**	**Key molecular features/Characteristics**	**Potential targeted therapy**
Basal-like 1 [6, 11]	High expression of DDR genes (e.g., ATR/BRCA pathway) and proliferation genes; high genomic instability. Frequently associated with *BRCA1/2* mutations.	PARP inhibitors, platinum-based chemotherapy (cisplatin, carboplatin), and DNA repair pathway inhibitors (e.g., ATM, DNAPK inhibitors).
Basal-like 2 [6, 11, 26]	Activation of growth factor signaling pathways (e.g., EGFR, MET, IGF-1R, Wnt/β-catenin); enrichment of glycolysis and gluconeogenesis pathways; expression of myoepithelial markers.	Growth factor inhibitors (e.g., lapatinib, gefitinib, cetuximab), MAPK pathway inhibitors, mTOR inhibitors, and CDK4/6 inhibitors.
Mesenchymal [6, 11]	High expression of genes involved in EMT, motility, adhesion, and stem cell pathways (e.g., Wnt/β-catenin, TGF-β, VEGF, mTOR, PI3K/AKT). Exhibits sarcoma-like tissue characteristics and is associated with drug resistance.	Src inhibitors (Dasatinib), PI3K/mTOR inhibitors, inhibitors targeting EMT pathways, Wnt pathway inhibitors, and retinoic acid derivatives.
Luminal androgen receptor [6, 10, 11, 18]	High expression of AR and AR-activated signaling (e.g., steroid biosynthesis, androgen/estrogen metabolism); frequently exhibits *PIK3CA* mutations.	Anti-AR therapies (e.g., bicalutamide, enzalutamide, abiraterone), PI3K/AKT/mTOR pathway inhibitors, and potentially CDK4/6 inhibitors (ribociclib, palbociclib).
Immunomodulatory [6, 11, 14, 18]	High levels of TILs; enriched for immune signaling pathways (T-cell receptor, cytokine signaling); high expression of immune checkpoints (PD-1/PD-L1).	ICIs such as anti-PD-1 (pembrolizumab) or anti-PD-L1 (atezolizumab) agents, and potentially anti-CTLA-4 inhibitors.

AKT: protein kinase B; AR: androgen receptor; ATM: ataxia-telangiectasia mutated; ATR: ataxia telangiectasia and Rad3-related; CDK: cyclin-dependent kinase; CTLA-4: cytotoxic T-lymphocyte-associated protein 4; DDR: DNA damage response; EGFR: epidermal growth factor receptor; EMT: epithelial-to-mesenchymal transition; ICIs: immune checkpoint inhibitors; IGF-1R: insulin-like growth factor 1 receptor; MAPK: mitogen-activated protein kinase; MET: mesenchymal-epithelial transition; mTOR: mammalian target of rapamycin; PI3K: phosphoinositide-3 kinase; TGF-β: transforming growth factor beta; TILs: tumor-infiltrating lymphocytes; VEGF: vascular endothelial growth factor.

Adding to the Lehmann and Burstein frameworks, the Fudan University Shanghai Cancer Center (FUSCC) classification system has been pivotal in translating transcriptomic subtyping directly into clinical practice. Developed primarily from Asian cohorts, the FUSCC system categorizes TNBC into four therapeutic subtypes: immunomodulatory (IM), LAR, mesenchymal-like (MES), and basal-like immune-suppressed (BLIS) [30]. The clinical applicability of this framework was prospectively validated in the phase Ib/II FUTURE umbrella trial. By matching heavily pretreated metastatic TNBC patients to targeted therapies based on their FUSCC subtype and genomic biomarkers, the trial achieved a remarkable objective response rate (ORR) of 29.0%; notably, the IM subtype achieved a 52.6% ORR when treated with anti-PD-1 therapy plus nab-paclitaxel [31, 32]. Building on this success, the phase II FUTURE-SUPER trial evaluated first-line, subtyping-based therapies, demonstrating a significantly prolonged median progression-free survival (PFS) (11.3 months) compared to standard chemotherapy alone (5.8 months) [33]. These trials provide compelling evidence that prospective molecular subtyping can be successfully translated into therapeutic decision-making for metastatic TNBC.

## Emerging therapies and research breakthroughs

### Antibody-drug conjugates

**Figure 3:**
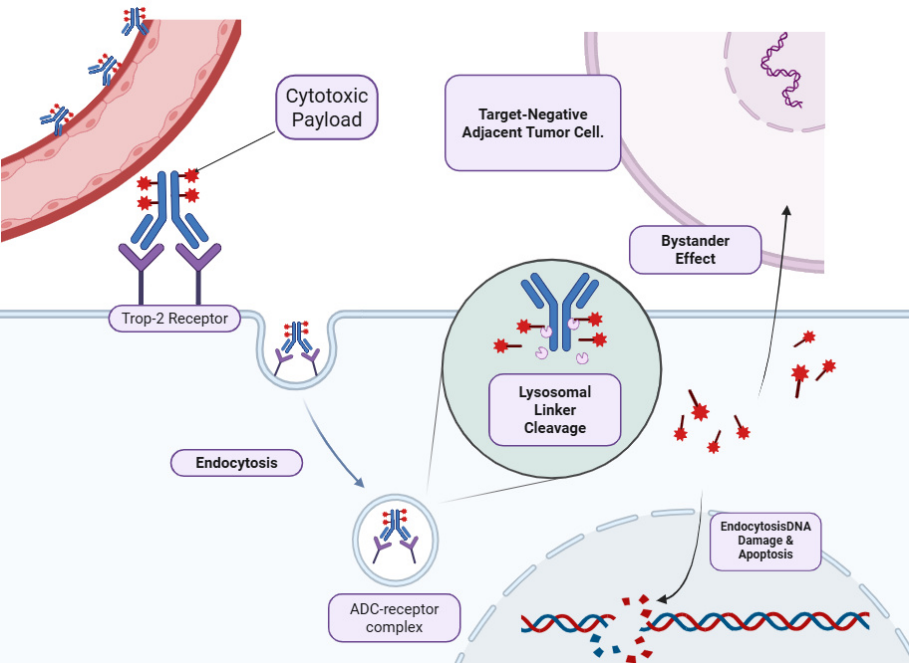
**Mechanism of action of Trop-2-directed antibody-drug conjugates (ADCs) in triple-negative breast cancer (TNBC).** The ADC (e.g., sacituzumab govitecan) is composed of a humanized monoclonal antibody targeting the trophoblast cell-surface antigen 2 (Trop-2) bound to a highly potent cytotoxic payload (e.g., the topoisomerase I inhibitor SN-38) via a hydrolyzable linker [11]. Upon binding to Trop-2 receptors—which are highly overexpressed on the TNBC cell surface—the ADC-receptor complex is internalized into the cell via endocytosis [36]. Subsequent acidification within the intracellular endolysosomal system triggers the cleavage of the linker, releasing the active SN-38 payload into the cytoplasm [11]. The payload translocates to the nucleus, where it inhibits DNA ligation, ultimately causing double-strand DNA breaks and cellular apoptosis [11]. Crucially, the membrane-permeable nature of the released payload allows it to diffuse out of the primary targeted cell to exert a potent “bystander effect”, effectively eradicating adjacent tumor cells even if they lack Trop-2 expression [13, 37]. Created in BioRender. Alobaidi, A. (2026) https://BioRender.com/cp5r0xp.

ADCs are targeted chemotherapies composed of a monoclonal antibody (mAb) linked to a cleavable linker and a potent cytotoxic payload. The mAb specifically recognizes and binds to an antigen overexpressed on the tumor cell surface, allowing for the selective internalization and release of the cytotoxic agent at the tumor site [19, 34, 35] (Figure 3).

SG (Trodelvy) was the first ADC approved for TNBC [11, 22]. It targets Trop-2 (expressed in > 90% of TNBCs) and delivers the topoisomerase-I payload SN-38 [19, 27, 35]. The clinical impact of SG for metastatic TNBC was demonstrated in the phase III ASCENT trial (NCT02574455) [29, 34], where it significantly outperformed single-agent chemotherapy in pre-treated patients [19, 28, 29]. SG yielded a median PFS of 5.6 months and a median OS of 12.1 months versus 1.7 months (PFS) and 6.7 months (OS) with single-agent chemotherapy [19, 28, 29]. Other ADCs in development include trastuzumab deruxtecan (T-DXd) targeting HER2 with a topoisomerase I payload and a high drug-to-antibody ratio (T-DXd delivers a potent topoisomerase I inhibitor payload) that produces a membrane-permeable “bystander effect,” killing adjacent cells regardless of high HER2 expression [10, 19, 34], thereby benefiting a subgroup of patients historically considered HER2-negative [22, 34]. Other promising ADCs in development include datopotamab deruxtecan (Dato-DXd), another Trop-2 targeting ADC utilizing a different payload [5, 13], and ladiratuzumab vedotin (LV), which targets the zinc transporter protein LIV-1 and delivers a microtubule inhibitor. Ongoing research is actively testing ADC combinations with targeted agents (e.g., SG + PARP inhibitors such as talazoparib) and with ICIs to enhance antitumor activity and exploit tumor vulnerabilities [6, 19, 27, 35].

Recent therapeutic developments reported in 2025 highlight the rapid evolution of biomarker-enriched trials and novel ADC combination strategies. For instance, the I-SPY2.2 trial utilized a novel neoadjuvant sequential therapy platform to evaluate the Trop-2-directed ADC Dato-DXd combined with the PD-L1 inhibitor durvalumab; this combination successfully graduated to phase 3 trials specifically for the “immune-positive” BC subtype [13]. Consequently, major phase 3 trials such as TROPION-Breast02 and TROPION-Breast03 are actively investigating Dato-DXd in the first-line metastatic and post-neoadjuvant settings, respectively [13]. Furthermore, clinical trial designs are increasingly relying on real-time liquid biopsies. Studies like the c-TRAK TN and ZEST trials are currently utilizing dynamic circulating tumor DNA (ctDNA) monitoring to guide the escalation of targeted therapies or ICIs, representing a shift toward highly personalized, adaptive treatment regimens [6].

### Combination and immunomodulatory strategies

TNBC’s aggressive nature, heterogeneity, and propensity for chemo-resistance have necessitated the exploration of synergistic combination strategies that leverage the IM effects of various therapeutic agents. Combining ICIs, specifically PD-1/PD-L1 inhibitors such as pembrolizumab and atezolizumab, with chemotherapy serves to raise tumor immunogenicity and has resulted in significantly higher neoadjuvant pCR rates [6, 19, 35]. Furthermore, preclinical and early clinical data support the use of ICI in combination with PARP inhibitors, as PARP inhibition can upregulate PD-L1 expression and subsequent tumor immunosuppression, which may be circumvented by PD-1/PD-L1 blockade, thus potentially enhancing efficacy [35, 38]. Combinations involving ADCs plus ICIs and ADCs plus targeted agents are also under active investigation to leverage complementary anti-tumor mechanisms [6, 19, 27].

### Other targeted pathways

The PI3K/AKT/mTOR pathway is also highly attractive due to its frequent activation (up to one-third of cases) in TNBC, promoting survival and chemoresistance; AKT inhibitors like capivasertib and ipatasertib, and PI3K inhibitors like alpelisib, are currently in trials [19, 26, 35]. AR is a potential target in the LAR molecular subtype, which constitutes 20–40% of TNBCs, with AR antagonists (e.g., enzalutamide, bicalutamide) being investigated, sometimes in combination with PI3K inhibitors or CDK4/6 inhibitors [19, 27, 35]. Research actively shows that detailed molecular profiling is now enabling a precision medicine approach, guiding the use of targeted therapies for specific subtypes and revealing new vulnerabilities for drug development [4, 13, 24, 27]. Despite a strong preclinical rationale, inhibitors of the PI3K/AKT/mTOR pathway have not yet been approved for TNBC and are not currently recommended by major clinical guidelines (such as ASCO, ESMO, or NCCN) for routine TNBC management [22, 26]. While the AKT inhibitor capivasertib is FDA-approved for HR+/HER2– BC, preliminary results from the phase III CAPItello-290 trial evaluating its efficacy in advanced TNBC were not satisfactory [22]. Currently, guideline-approved targeted therapies for metastatic TNBC are limited to pembrolizumab for PD-L1-positive disease (NCCN, ESMO, ASCO), PARP inhibitors such as olaparib and talazoparib for germline *BRCA1/2* mutations (NCCN, ESMO), and the ADC SG in the second-line setting and beyond [5].

### AI, radiomics, and the future of personalized care

The future trajectory of TNBC management is being reshaped by the convergence of advanced emerging technologies such as artificial intelligence (AI) and machine learning (ML) [9, 13, 22, 24]. AI methodologies, especially radiomics applied to imaging modalities like dynamic contrast-enhanced magnetic resonance imaging (MRI) and ultrasound, offer a non-invasive means to elucidate molecular and anatomical characteristics often imperceptible to the human eye [9, 10, 14, 15]. These computational approaches have demonstrated promising performance in both the differential diagnosis of TNBC and the early, pre-treatment prediction of pCR to NACT, thereby offering a crucial platform for personalized clinical decision-making and early therapy adjustment [6, 9, 15, 24]. This technological integration accelerates the refinement of biomarker-based approaches, moving treatment beyond the traditional three negative receptor definition toward strategies guided by inherent molecular subtype classifications (e.g., LAR, BLIS, MES) derived from genomic and transcriptomic analyses [5, 13, 15, 18]. By integrating multi-omics data, scientists can identify highly specific vulnerabilities to guide the selection of appropriate targeted therapies, ultimately ensuring a more effective and tailored therapeutic strategy for individual TNBC patients [13, 18].

Traditional diagnostic methods, such as tissue biopsies, are inherently invasive and often fail to capture the full spectrum of spatial and temporal tumor heterogeneity [39]. To significantly improve diagnostic specificity and reduce invasiveness, liquid biopsy can be used [7, 40]. Liquid biopsy can analyze ctDNA and circulating tumor cells (CTCs) from a simple blood draw, and has emerged as a powerful, non-invasive alternative. Liquid biopsies allow for real-time, dynamic monitoring of tumor evolution and the early detection of minimal residual disease months before clinical relapse [7, 40]. Furthermore, integrating AI and radiomics with standard imaging modalities (such as MRI and ultrasound) provides a non-invasive method to extract deep molecular and anatomical features, improving the differential diagnosis and early prediction of treatment response without the need for repeated invasive procedures [41].

### Theranostics and nanomedicine platforms

To seamlessly integrate both diagnostic and therapeutic capabilities within a single TNBC intervention, the field is rapidly advancing toward “theranostic” nanomedicine platforms [42]. Theranostics utilize engineered nanoparticles designed to simultaneously detect malignant cells and deliver targeted therapies [43]. For example, radionuclide-functionalized nanoparticles can be employed for high-resolution molecular imaging [such as PET (positron emission tomography) or SPECT (single photon emission computed tomography)] while simultaneously delivering targeted radionuclide therapy directly to the TNBC microenvironment [43]. Similarly, conjugated polymer nanoparticles and gold nanoparticles serve as dual-action platforms; they act as contrast agents for real-time fluorescent tumor imaging while concurrently generating cytotoxic reactive oxygen species or localized hyperthermia upon near-infrared light irradiation, thereby enabling precise, targeted photodynamic and photothermal therapies against TNBC cells [44, 45]. These integrated approaches offer a promising strategy to maximize local drug concentrations while minimizing off-target systemic toxicity [46].

## Challenges, disparities, and clinical gaps

Racial, socioeconomic, and healthcare system disparities affect outcomes in TNBC, which disproportionately impacts younger, premenopausal women and those from minority populations, particularly non-Hispanic Black women [4, 13]. Non-Hispanic Black women have nearly double the incidence of TNBC compared with White women and experience significantly higher mortality, a pattern that persists even after adjusting for socioeconomic status and stage at diagnosis [4, 13, 26]. This elevated risk arises from a complex interaction of factors, including higher prevalence of germline *BRCA1* and *BRCA2* mutations and distinct genetic and epigenetic features in Black women [2, 6, 13]. Non-biological factors further worsen outcomes [13, 26]. Barriers such as delayed or limited access to screening, financial obstacles to completing treatment, and a later stage at diagnosis are common among minority groups [5, 13, 26]. TNBC is also frequently identified as an interval cancer because of its rapid growth and the higher mammographic density often present in younger women [4, 9]. Emerging multi-omics data from East Asian cohorts reveal distinct biological landscapes. Genomic profiling of Chinese TNBC patients has demonstrated a significantly higher prevalence of the LAR subtype (23%) compared to African American (9%) or Caucasian (12%) cohorts. Furthermore, East Asian TNBC tumors exhibit a higher frequency of PIK3CA mutations (18% vs. 10%) and more frequent somatic copy-number gains on chromosome 22q11 compared to Western cohorts from The Cancer Genome Atlas (TCGA) [30]. Similarly, analyses of Japanese TNBC cohorts have identified significant differences in the amplification frequencies of specific driver genes, such as *MYC* and *PTK2*, compared to non-Asian databases [47]. These ethnic and geographic genomic variations highlight a clear need to integrate Asian-led clinical evidence into precision oncology strategies. Additionally, racial and ethnic minorities remain significantly underrepresented in clinical trials, which limits the generalizability of study findings and restricts access to emerging therapies [5, 13, 48, 49]. These systemic disadvantages amplify both psychological distress and financial toxicity, including income loss and treatment-related financial burdens, especially among young and Black women undergoing chemotherapy [49].

Targeted research, health equity initiatives, and personalized care strategies are essential to reduce these disparities and improve outcomes [2, 4, 13, 48]. A major obstacle to developing widely effective therapies is the substantial molecular and biological heterogeneity of TNBC. This emphasizes a critical need for reliable predictive biomarkers. Currently, biomarker-driven treatment selection is limited [6, 15, 24, 27]. Germline *BRCA1* and *BRCA2* mutations identify a small subset responsive to PARP inhibitors, and PD-L1 expression is used to guide ICI therapy in the metastatic setting, although its predictive value varies across clinical trials. Ongoing research aims to integrate multi-omics data and incorporate biomarkers such as TILs, TMB, and molecular subtyping to develop more comprehensive predictive models [6, 11, 13–15, 19].

Future improvements in TNBC outcomes depend on advancing precision medicine and technology to enable earlier detection and individualized intervention [13, 22, 24]. Liquid biopsy, particularly ctDNA, provides an opportunity to detect minimal residual disease and molecular recurrence months before clinical symptoms appear. This creates valuable lead time for intensifying therapy and optimizing post-neoadjuvant risk stratification [5, 6, 15, 39]. Achieving equitable progress also requires addressing persistent racial and socioeconomic disparities [4, 13, 26]. Precision oncology advances, including genomic and molecular subtyping, must be paired with improved access to specialized therapies such as ADCs and ICIs, expanded inclusion of minority patients in clinical trials, and systemic efforts to reduce the significant financial and psychological burdens experienced by those diagnosed with TNBC [5, 13, 48, 49]. A comprehensive and equitable model is essential to ensure that all women affected by this aggressive disease subtype benefit from emerging scientific and clinical progress [4, 13, 18, 22].

Despite profound advances in defining the distinct molecular subtypes of TNBC, these classifications remain largely theoretical and have not yet been incorporated into routine clinical practice or current TNBC treatment guidelines [15, 36]. Routine clinical implementation is significantly hindered by the high cost and limited accessibility of the RNA-sequencing required for tumor profiling, as well as a lack of consensus among the various classification systems. Consequently, outside of clinical trials, TNBC is largely treated as a single disease entity, with precision treatment decisions relying primarily on a few accessible biomarkers, namely PD-L1 immunohistochemistry staining and germline *BRCA1/2* mutational status [15].

Beyond traditional nanoparticles, the development of functional polymers and metal-organic frameworks (MOFs) has introduced highly versatile nanoplatforms capable of integrating diagnosis and therapy. Due to their unique two- and three-dimensional architectures, high specific surface area, and tunable pore dimensions, MOFs and functional polymers are being extensively utilized for targeted drug delivery, biosensing, and therapeutic monitoring across a variety of complex systemic diseases [50]. Adapting these advanced polymeric and MOF platforms to oncology holds immense promise for overcoming chemoresistance. For instance, functional MOF-based nanocarriers have recently demonstrated the ability to effectively ablate primary breast tumors and significantly inhibit lung metastasis in TNBC models while carrying diagnostic imaging agents [50]. Expanding the use of these reactive and functional polymers could provide the precise, multi-target delivery systems needed to overcome TNBC’s severe heterogeneity.

## Conclusions

The future of TNBC care requires a shift from empiric chemotherapy toward highly personalized strategies informed by the disease’s complex and heterogeneous biological ecosystem [5, 19]. Therapeutic progress will depend on rationally designed combination regimens, such as integrating ADCs, ICIs, and PARP inhibitors, to overcome acquired resistance [6, 19]. This approach necessitates rigorous, multi-level clinical trials to define optimal sequencing and timing of these agents [17, 34, 35]. Achieving truly individualized care will require moving beyond single-biomarker selection toward multi-omics and transcriptomic integration to refine molecular subtyping and match therapies to tumor-specific vulnerabilities [13, 15, 24]. Equally essential is the commitment to health equity. Eliminating racial and socioeconomic disparities demands expanded access to screening, intentional inclusion of underrepresented minorities in clinical trials, and comprehensive strategies to reduce financial toxicity, particularly for younger and Black women who bear a disproportionate burden of TNBC [5, 13]. Emerging technologies, including AI and ML applied to radiomics and histopathology, hold significant promise for enhancing diagnosis and predicting treatment response [13]. Liquid biopsy and dynamic ctDNA monitoring offer new avenues for early, non-invasive detection of recurrence and real-time adaptation of therapy, marking a pivotal step toward precision-guided and equitable TNBC management [13]. TNBC remains one of the most challenging malignancies in oncology, not because it is poorly understood, but because its biology is aggressive, heterogeneous, and highly adaptable. Yet it has also become one of the most important drivers of innovation, transforming from a subtype defined by the absence of therapeutic targets into one shaped by emerging opportunities in immunotherapy, DNA repair inhibition, and ADCs.

## Abbreviations

ADCs: antibody-drug conjugates
AI: artificial intelligence
AKT: protein kinase B
BC: breast cancer
BLIS: basal-like immune-suppressed
CAFs: cancer-associated fibroblasts
ctDNA: circulating tumor DNA
Dato-DXd: datopotamab deruxtecan
DSBs: double-strand breaks
ECM: extracellular matrix
ER: estrogen receptor
FUSCC: Fudan University Shanghai Cancer Center
HER2: human epidermal growth factor receptor 2
HR: hormone receptor
HRD: homologous recombination deficiency
ICIs: immune checkpoint inhibitors
IM: immunomodulatory
LAR: luminal androgen receptor
mAb: monoclonal antibody
MES: mesenchymal-like
ML: machine learning
MOFs: metal-organic frameworks
MRI: magnetic resonance imaging
mTOR: mammalian target of rapamycin
NACT: neoadjuvant chemotherapy
ORR: objective response rate
OS: overall survival
PARP: poly(ADP-ribose) polymerase
pCR: pathological complete response
PD-L1: programmed death ligand 1
PFS: progression-free survival
PI3K: phosphatidylinositol-3 kinase
PR: progesterone receptor
SG: sacituzumab govitecan
TAMs: tumor-associated macrophages
T-DXd: trastuzumab deruxtecan
TILs: tumor-infiltrating lymphocytes
TMB: tumor mutational burden
TME: tumor microenvironment
TNBC: triple-negative breast cancer

## Declarations

### Copyright

© The Author(s) 2026.
